# Assigning Main Orientation to an EOH Descriptor on Multispectral Images

**DOI:** 10.3390/s150715595

**Published:** 2015-07-01

**Authors:** Yong Li, Xiang Shi, Lijun Wei, Junwei Zou, Fang Chen

**Affiliations:** 1 Beijing University of Posts and Teles., School of Electronic Engineering, Rd. Xitucheng 10#, Beijing 100876, China; E-Mails: 2013140640@bupt.edu.cn (X.S.); weilijun@bupt.edu.cn (L.W.); buptzjw@bupt.edu.cn (J.Z.); 2 Beijing Anzhen Hospital, Capital Medical University and Beijing Institute of Heart Lung and Blood Vessel Disease, Beijing 100029, China; E-Mail: azchenfang@163.com

**Keywords:** keypoint, main orientation, EOH, descriptor

## Abstract

This paper proposes an approach to compute an EOH (edge-oriented histogram) descriptor with main orientation. EOH has a better matching ability than SIFT (scale-invariant feature transform) on multispectral images, but does not assign a main orientation to keypoints. Alternatively, it tends to assign the same main orientation to every keypoint, e.g., zero degrees. This limits EOH to matching keypoints between images of translation misalignment only. Observing this limitation, we propose assigning to keypoints the main orientation that is computed with PIIFD (partial intensity invariant feature descriptor). In the proposed method, SIFT keypoints are detected from images as the extrema of difference of Gaussians, and every keypoint is assigned to the main orientation computed with PIIFD. Then, EOH is computed for every keypoint with respect to its main orientation. In addition, an implementation variant is proposed for fast computation of the EOH descriptor. Experimental results show that the proposed approach performs more robustly than the original EOH on image pairs that have a rotation misalignment.

## 1. Introduction

Keypoint and descriptor techniques have been widely applied in computer vision or pattern recognition. Applications include stereo vision, 3D scene reconstruction, human activity recognition, *etc*. Keypoints are often matched by computing the distance of their associated descriptors. The matching ability of descriptors is measured with the repeatability and distinctiveness, and in practice, a trade-off is often made between them. On single spectral images, SIFT [1] and its variants with post-processing techniques (e.g., RANSAC) have witnessed many successful applications. On multi-sensor (multispectral) images, SIFT descriptors generate few correct mappings. Recently, the edge-oriented histogram (EOH) [2] was proposed, which utilizes only edge points and five bins for computing descriptors. EOH has a better matching performance on multispectral images than SIFT, but does not assign a main orientation to keypoints, which limits its application to images containing translation misalignment.

### 1.1. Related Work

Salient points have been widely used in a variety of fields, including object tracking, image fusion, intelligent navigation, *etc*. [3–9]. Many keypoint and descriptor detection techniques have been proposed for single spectral images. Lowe [1] proposed SIFT detecting keypoints invariant to scale and rotation. The keypoints are defined to be the extrema of the difference of Gaussians (DOG). The local gradient pattern around a keypoint with respect to an assigned main orientation is computed as its descriptor. Bay *et al.* [10] proposed SURF (speeded-up robust features). SURF has the same repeatability and distinctiveness as SIFT, but is computed faster than SIFT. Alahi *et al.* [11] proposed fast retina keypoint (FREAK). FREAK is a cascade of binary strings computed by comparing image intensities over a retinal sampling pattern. Ambai and Yoshida [12] proposed compact and real-time descriptors (CARD). Compared with SIFT and SURF, CARD can be computed rapidly utilizing lookup tables to extract histograms of oriented gradients. Other descriptors include ORB [13] and PCA-SIFT [14].

The above descriptors are devised for single-sensor images and yield a good matching performance on such images. Recently, multispectral systems became an attractive research topic, since they provide a rich representation of scene with images taken by different sensors [15]. Barrera *et al.* proposed an imaging system for computing depth maps from color and infrared images [16]. Stereo vision can be accomplished by keypoint matches. However, the descriptors, such as SIFT, SURF and ORB, are computed by utilizing the gradient pattern, which may revert on multispectral images [17,18], and hence, their performance deteriorates [19]. Since the computing gradient is a linear operation of original image intensities, the matching ability of descriptors relies on the linear relationship between image intensities.

Three factors contribute to the decrease of matching ability: the repeatability of keypoints, the accuracy of main orientation and the repeatability/distinctiveness of descriptors. From the perspective of descriptors, many techniques have been proposed to adapt descriptors of SIFT/SURF to multispectral images. Chen *et al.* [18] proposed the partial intensity invariant feature descriptor (PIIFD), which uses gradient orientation instead of direction. The gradient orientation is limited within [0, *π*), and PIIFD can register poor-quality multi-modal retinal image pairs. Saleem and Sablatnig [20] proposed NG-SIFT, which computes descriptors using a normalized gradient. NG-SIFT outperforms SIFT on image pairs of a visible image and a near-infrared image. Dellinger *et al.* [21] proposed SAR-SIFT for SAR images. SAR-SIFT uses a new gradient computation method, gradient by ratio (GR), which is robust to speckle noise, so that it can perform better on SAR images than SIFT. Hossain *et al.* [17] proposed the symmetric-SIFT algorithm for multi-modal image registration. It overcomes the problem that gradient direction could be inverted in different sensors.

Aguilera *et al.* [2] proposed the edge-oriented histogram (EOH). Unlike SIFT, EOH exploits only edge points in local windows rather than all pixels, since in general, edges are more likely repeatable and, hence, tending to be reliable between multi-sensor images. For an edge pixel, five responses are computed with filters designed in [22]. Edge points are detected by the Canny detector [23]. Note that the first four filters are directional derivatives, and the fifth filter is “no direction”. The problem with EOH is that it does not assign a main orientation to keypoints, which amounts to assuming that the main orientation for all keypoints takes on the same value, e.g., 0°. When the misalignment does not contain a rotation component, EOH works pretty well [2], but this limits the application of EOH to translation only. In real applications, image pairs taken from different views often contain a rotation component in the misalignment, and rotation invariant descriptors are hence desired or necessitated.

### 1.2. Proposed Method

To adapt EOH to dealing with rotation, we propose assigning a main orientation to keypoints for EOH computation. The main orientation makes EOH invariant to both translation and rotation and, hence, invariant to similarity transformation and partially invariant to affine transformations [1]. Note that the rotation contained in the misalignment is unknown and by no means can one obtain it before building keypoints.

Gauglitz *et al.* [24] proposed two orientation assignment methods. One method is to utilize the center-of-mass (COM), which is suitable for corners, and the other one is to utilize the histogram of intensities (HOI). Both COM and HOI are more suitable for single-spectral images, since they implicitly use the linear relationship of image intensities. This work utilizes the main orientation provided by PIIFD [18]. When a main orientation is assigned to a keypoint, the computation of its associated EOH descriptor needs interpolation at fractional pixels. Bilinear interpolation is applied to compute the response of the five filters used by EOH.

The rest of the paper is arranged as follows. Section 2 discusses assigning main orientation to keypoints and computing descriptors for keypoints relative to the main orientation, Section 3 gives the matching scheme for keypoints, Section 4 presents experimental results comparing the matching performance, and Section 5 concludes this paper.

## 2. Assigning Main Orientation to the Keypoint and Compute Descriptor

This section discusses assigning main orientation to keypoints, then the descriptor is computed for every keypoint with respect to the assigned main orientation.

### 2.1. Why a Main Orientation is Needed for Keypoints

Let *I_r_*(*x*, *y*) and *I_t_*(*x*, *y*) denote the reference and the test image to be registered. SIFT [1] calculates the histogram of gradient orientation and finds its peak in a local window to serve as the main orientation. Chen *et al.* [18] considered the problem of gradient and/or region reversal and square gradient (*G_x_,G_y_*) by:

(1) $$\left[\begin{array}{c} G_{s,x}\\ G_{s,y} \end{array}\right]=\left[\begin{array}{c} G_{x}^{2}-G_{y}^{2}\\ 2G_{x}G_{y} \end{array}\right]$$

and then smoothed the squared gradient by convolving it with an average filter *h_σ_*,

(2) $$\left[\begin{array}{c} \bar{G_{s,x}}\\ \bar{G_{s,y}} \end{array}\right]=\left[\begin{array}{c} G_{s,x}⊗h_{\sigma }\\ G_{s,y}⊗h_{\sigma } \end{array}\right]$$

The main orientation is calculated as follows,

(3) $$\phi =\frac{1}{2}\{\begin{array}{ll} \tan^{-1}(\bar{G_{s,y}}/\bar{G_{s,x}})+\pi , & \bar{G_{s,x}}\geq 0\\ \tan^{-1}(\bar{G_{s,y}}/\bar{G_{s,x}})+2\pi , & \bar{G_{s,x}}<0\cap \bar{G_{s,y}}\geq 0\\ \tan^{-1}(\bar{G_{s,y}}/\bar{G_{s,x}}), & \bar{G_{s,x}}<0\cap \bar{G_{s,y}}<0 \end{array}$$

A careful derivation shows that the main orientation *ϕ* and the traditional gradient (*G_x_*, *G_y_*) roughly have the following relationship. Let *θ* = *atan*2 (*G_y_*, *G_x_*) be the gradient direction falling in [−*π*, *π*]. *atan*2 is the four-quadrant inverse tangent for a gradient (*G_x_*, *G_y_*), giving the actual gradient direction for (*G_x_*, *G_y_*). In mathematics, it differs from tan^−1^ in that the range of tan^−1^ is $\left(-\frac{\pi }{2},\frac{\pi }{2}\right)$, while the range of *atan*2 is (−*π*, *π*)*. θ* is mapped to [0, *π*] by setting *θ* = *mod*(*θ*, *π*), *i.e.*,

(4) $$\theta =\{\begin{array}{cc} \theta , & \theta \in [0,\pi ]\\ \theta +\pi , & \theta \in [-\pi ,0] \end{array}$$

Then:

(5) $$\phi =\{\begin{array}{cc} \theta +\pi /2, & \theta \in [0,\pi /2]\\ \theta -\pi /2, & \theta \in [\pi /2,\pi ] \end{array}$$

Equations (4) and (5) indicate that a gradient direction *θ* and its reversal direction *θ*±*π* will contribute to the same main orientation bin.

EOH is applied to *I_r_*(*x*, *y*) and *I_t_*(*x*, *y*) to detect keypoints and descriptors. Let $K_{t}^{i}$, *i* = 1, …, *N_t_*, denote the *i*-th keypoint on the test image *I_t_*(*x*, *y*), and $K_{r}^{j}$, *j* = 1, …, *N_r_*, denote the *j*-th keypoint on the reference image *I_r_*(*x*, *y*). Let $f_{t}^{i}$, *i* = 1, …, *N_t_*, denote the descriptor of $K_{t}^{i}$, and $f_{r}^{j}$, *j* = 1, …, *N_r_*, denote the descriptor of $K_{r}^{j}$. Note that both EOH [2] and PIIFD [18] employ the extrema of DOG as keypoints, which was proposed in SIFT. When detecting keypoints, *σ_n_* = 0.5 is the standard deviation of the Gaussian function used for nominal smoothing of an input image. The threshold on the ratio of principle curvatures is set to 10, the default value in SIFT [1].

This work assigns the main orientation computed with PIIFD to keypoints $K_{t}^{i}$, *i* = 1, …, *N_t_* and $K_{r}^{j}$, *j* = 1, …, *N_r_*, then computes EOH descriptors. Like SIFT, the process of building keypoint matches includes four steps: (1) detect keypoints to be the extrema of DOG as proposed by SIFT; (2) assign to every keypoint main orientation computed with PIIFD; (3) compute the EOH descriptor for each keypoint with respect to its main orientation. Edge points are detected by the Canny operator, and all parameters of the Canny detectors are set to default values used by the MATLAB implementation, except that the standard deviation of the Gaussian filter *σ* is set to three, like in the original EOH [2]. The high threshold is defined to be the gradient magnitude ranked as the top 30%, and the low threshold is defined to be 40% of the high threshold. Interpolation is needed here to obtain pixel values at fractional pixels. Finally, (4) match the keypoint with the computed descriptor.

### 2.2. Compute the Descriptor for Keypoint with the Main Orientation

**Figure 1:**
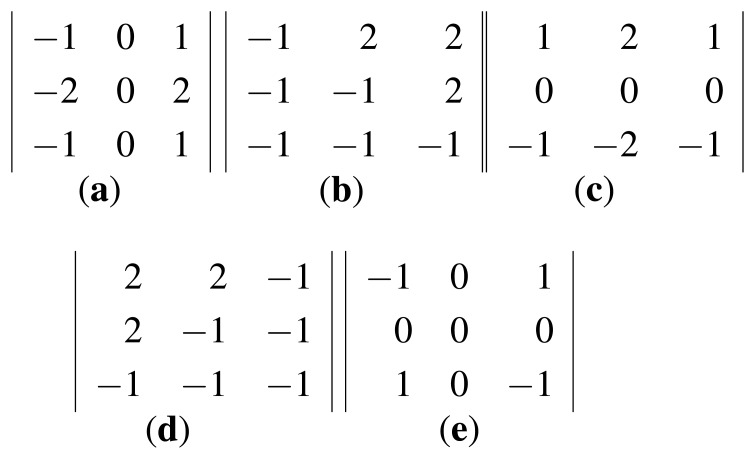
The five filters used in [2,22]. The filters compute directional derivatives in 0°, 45°, 90°, 135° and the non-direction showed in (**a–d**) and (**e**) respectively. (**a–d**) are the direction filters and (**e**) is the non-direction filter.

The EOH computes the gradient orientation at each edge pixel with the following five filters. These filters correspond to the 0°, 45°, 90°, 135° and non-direction, as shown in Figure 1. The filters shown in Figure 1a–d, are called direction filters, while the one shown in Figure 1e is called the non-direction filter. For a pixel, the filter giving the maximum response is defined to be the direction at the pixel. Formally, let *f_k_*(*x*, *y*),*k* = 0, 1, 2, 3,4, denote the mathematical representation of the five filters shown in Figure 1, then an edge pixel at (*x*, *y*) will contribute one to the bin defined by:

(6) $${\text{bin}}^{\text{EOH}}(x,y)=\arg\underset{k}{\max}|f_{k}(x,y)•I(x,y)|$$

where ‘•’ is the correlation between image and filter.

The four direction filters shown in Figure 1 are in fact the orientation partition used in PIIFD. SIFT employs eight orientations (bins) for computing descriptors, *i.e.*, for a pixel, its gradient orientation is quantized to eight bins with the center of each bin being 0°, 45°, 90°, 135°, 180°, 225°, 270°, 315°. PIIFD considers the gradient reverse and utilize mod-180° orientation. Specifically, let *α_x,y_* be the gradient orientation at (*x*, *y*). For SIFT, *α_x,y_* contributes to the bin:

(7) $${\text{bin}}^{\text{SIFT}}(\alpha_{x,y})=\text{round}\;(\frac{\alpha_{x,y}}{45^{\circ }})\%8$$

For PIIFD, it contributes to the bin:

(8) $${\text{bin}}^{\text{PIIFD}}(\alpha_{x,y})=\text{round}\;(\frac{\alpha_{x,y}}{22.5^{\circ }})\%8$$

Similar to SIFT, PIIFD uses eight bins in Equation (8). However, Equation (8) maps an orientation *α_x,y_* and *α_x,y_* + 180° to the same bin, *i.e.*, *bin*^PIIFD^(*α_x_,_y_*) = *bin*^PIIFD^(*α_x,y_* + 180°). PIIFD partitions [0, 2*π*] into 16 bins at first, with each bin covering 22.5°, and then “merges” two bins if their center angles differ by 180°.

Equation (7) also says that the centers of the first four orientation bins for SIFT are 0°, 45°, 90°, 135°, exactly the same as EOH bins. Thus,

(9) $${\text{bin}}^{\text{SIFT}}(\alpha_{x,y})\%4=bin^{\text{EOH}}(\alpha_{x,y})$$

**Figure 2:**
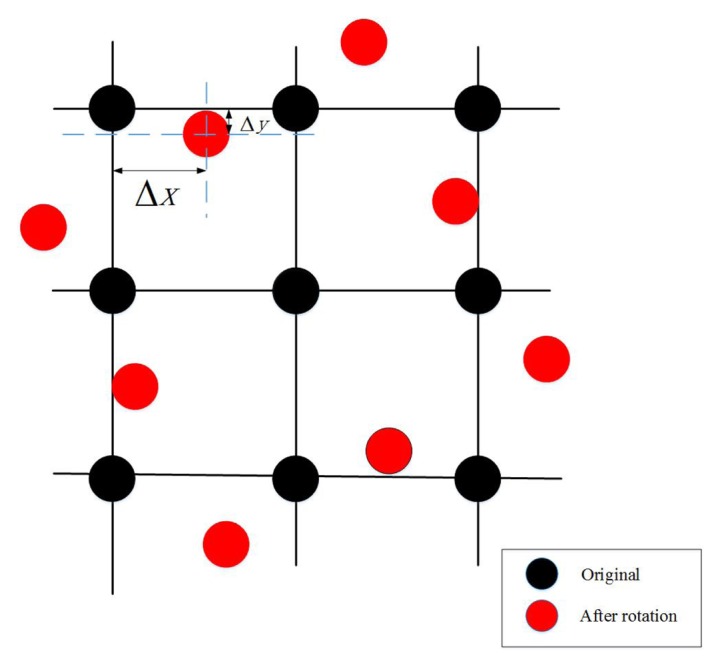
When the main orientation is assigned to a keypoint, the filters shown in Figure 1 ought to be rotated with respect to the main orientation. Black dots represent the integer pixel grid, and red dots are the fractional pixel locations, whosewhole values are used by the rotated filters.

When main orientation is assigned to a keypoint, we need to compute the maximum response of the five filters. The five filters are rotated by the amount of main orientation, and the rotated pixels for computing the filter response lie in a fractional grid, as shown in Figure 2. To obtain pixel values at the fractional grid, a bilinear interpolation is employed.

After the interpolation step, pixel values for computing the filter response are obtained. The filter giving the maximum response is defined to be the direction at this pixel and contributes to EOH descriptors. As in the original EOH [2], a local window of radius 50 is used for computing an EOH descriptor. Only edge pixels in the window contribute to the descriptor. Alternatively, we can skip the interpolation step and just utilize the gradient orientation at edge pixels, which is discussed in Section 2.3 as a variant of implementing EOH.

### 2.3. Variant Implementation of EOH

Computing the responses of five filters shown in Figure 1 can be speeded up by fast Fourier transform (FFT). We use *I_t_*(*x*, *y*) as an example and compute the filter responses with FFT. A similar process can be applied to *I_r_*(*x*, *y*). Let $I_{t}^{0}(x,y)$ denote the response of the zeroth filter (0°) applied to *I_t_*(*x*, *y*). Formally,

(10) $$I_{t}^{0}(x,y)=I_{t}(x,y)•f_{0}(x,y)=I_{t}(x,y)*f_{0}^{'}(x,y)$$

wherein ‘•’ denotes the correlation, which can be rewritten as the convolution of $f_{0}^{'}(x,y)$ and *I_t_*(*x*, *y*). $f_{0}^{'}(x,y)$ is the version of *f*_0_(*x*, *y*) flipped left-right and up-down, *i.e.*, $f_{0}^{'}(x,y)=f_{0}(-x,-y)$. The convolution $I_{t}(x,y)*f_{0}^{'}(x,y)$ can be quickly implemented with FFT.

The equivalence of four directional filter responses in EOH to the first four orientation bins in SIFT and the above discussed fast computation by FFT, motivate implementing EOH as follows. Define the directional gradient along the horizontal and vertical axes to be:

(11) $$\begin{array}{cc} G_{x}(x,y) & =I(x,y)•f_{0}(x,y)\\ G_{y}(x,y) & =I(x,y)•f_{2}(x,y) \end{array}$$

Note, the gradient computation in Equation (11) is nothing but the Sobel operator [25]. Once the directional gradient is obtained, its direction can be simply calculated by:

(12) $$\alpha (x,y)=\mathrm{atan}2(G_{y}(x,y),G_{x}(x,y))$$

Equation (11) together with Equation (9) gives the orientation bin to which every edge pixel contributes. By this means, we implement a variant of EOH, which can be understood from the adaptation of different descriptors.

- The variant ignores the non-direction bin, which in our experiments proved to have little effect on matching performance. See the analysis in Tables 1 and 2 in Section 4. Furthermore, the orientation bin computed with Equations (12), (11) and (9) may not be identical to that computed with Equation (6). See Section 4 for their matching performance comparison.
- NG-SIFT [20] utilizes the normalized gradient magnitude $\hat{\Omega }(x,y)=\sqrt{{\hat{F}}_{h}^{2}(x,y)+{\hat{F}}_{v}^{2}(x,y)}$ to compute the descriptor and does not distribute a pixel to neighboring spatial/orientational bins. See Equations (5)–(7) in [20]. EOH is similar to NG-SIFT from this aspect.
- The proposed variant of EOH utilizes the Sobel operator in Equation (11), and SIFT, PIIFD and NG-SIFT utilize the filters [−1, 0, 1] and [−1, 0, 1]*^T^* to calculate the horizontal and vertical directional derivatives.

## 3. Matching Keypoints with Descriptors

This section discusses matching keypoints with descriptors. The matching ability of descriptors is evaluated with the number of correct keypoint matches. To make a fair comparison for different descriptors, a simple matching approach suggested by SIFT is employed here. A reference keypoint $K_{r}^{j_{0}}$ is defined to be matched to a test keypoint $K_{t}^{i_{0}}$ if:

(13) $$D(f_{t}^{i_{0}},f_{r}^{j_{0}})<0.8\cdot D(f_{t}^{i_{0}},f_{r}^{j_{1}})$$

where *D*(·,·) is the Euclidean distance and $f_{r}^{j_{1}}$ is the second-closest neighbor to $f_{t}^{i_{0}}$. The ‘0.8’ in Equation (13) can be changed to 0.6, which means a tighter matching criterion giving fewer matched keypoints.

Equation (13) is the matching method suggested in the original SIFT [1]. Through Equation (13), a set of keypoint mappings can be established, which will be used to analyze the descriptor performance. See Section 4 for details. Note that post-processing techniques can be applied to keypoints and descriptors for removing outlier keypoint matches. Commonly-used techniques include RANSAC [26,27], its variant fast sample consensus (FSC) [28], *etc*. However, post-processing is to some extent independent of descriptor matching ability, and the resulting improvement ought to be excluded for comparing descriptors.

## 4. Experimental Results

This section presents the experimental results. Visual matching results are provided firstly, followed by the quantitative analysis on matching results. The proposed method is compared with the original EOH. Two datasets, EOIRand VS-long-wave infrared (LWIR), are used for investigating the matching performance. EOIR includes 87 image pairs captured by ourselves, 12 Landsat image pairs from NASA. The 87 image pairs include outdoor and indoor scenes with one image taken with visible light and the other taken with middle-wave infrared (MWIR) light. The 12 Landsat image pairs were downloaded from [29] with one taken with the visible band, e.g., Landsat 8 Band 3 Visible (0.53–0.59 µm), and the other taken with middle-wave light or the Thermal Infrared Sensor (TIRS), e.g., Landsat 8 Band 10 TIRS 1 (10.6–11.19 µm). Dataset VS-LWIR is from [2] containing 100 image pairs, one image taken with the visible bandwidth (0.4–0.7 µm) and the other taken with the long-wave infrared bandwidth (LWIR, 8–14 µm).

### 4.1. Visual Results

**Figure 3:**
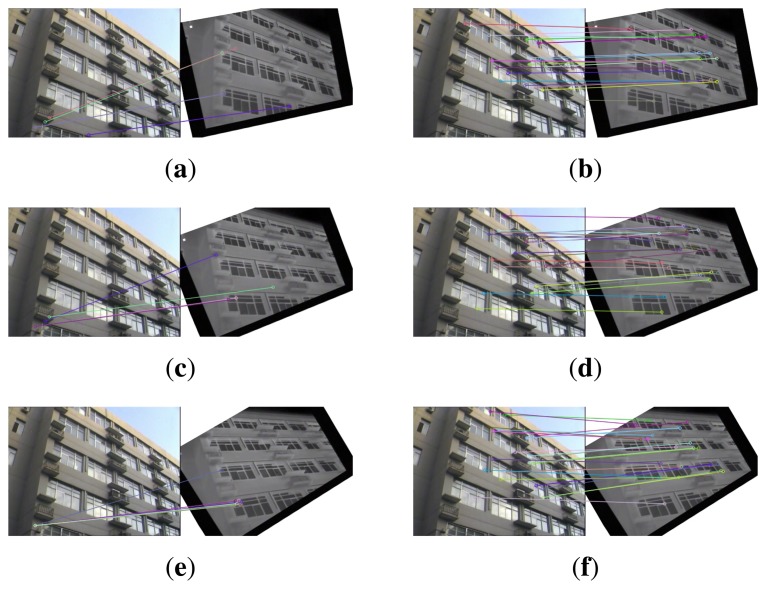
The matching performance under rotation. The test image is rotated by 10°, 20°, and 30° from top to bottom line. The left column is the result of EOH without main orientation. The right column is the result of EOH equipped with the partial intensity invariant feature descriptor (PIIFD) main orientation.

This section gives visual matching results. Figure 3 gives the keypoint matchings built with the original EOH without the main orientation and the proposed method. The visible image serves as the reference image, and the infrared image is used as the test image. The test image is rotated by 10°, 20° and 30°. Figure 3a,c,e show the matching result of EOH between the reference and the rotated test image by 10°, 20° and 30°, respectively. Due to the lack of main orientation, the keypoint matches built with the EOH contain very few or no correct matches. As a comparison, the proposed method provides sufficiently many correct matches in Figure 3b,d,f.

**Figure 4:**
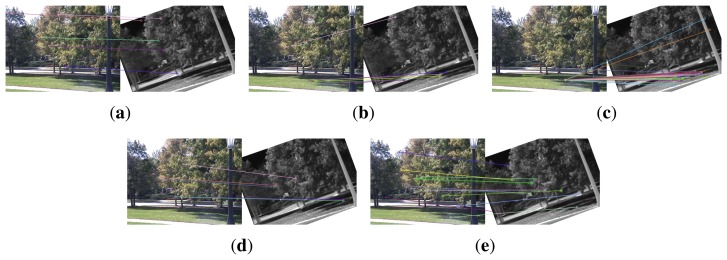
The matched keypoints built with descriptors. (**a**) The original EOH without main orientation; (**b**) the main orientation computed by SIFT, ranging from [0, 2*π*]; (**c**) the main orientation computed by center-of-mass (COM); (**d**) the main orientation computed by the histogram of intensities (HOI); (**e**) the proposed method that utilizes the main orientation computed by PIIFD. The test (IR) image is rotated by 20°.

Figure 4 shows the keypoint matches on an image pair from dataset EOIR built with EOH, EOH equipped with SIFT main orientation, with COM (center-of-mass) main orientation [24], with HOI (histogram of intensity) main orientation [24] and the proposed method. The infrared image is rotated by 20°. EOH provides five keypoint matches in Figure 4a, and three are visually correct. SIFT main orientation gives seven keypoint matches in Figure 4b, and four matches are visually correct. The COM and HOI main orientations do not give many correct matches, as shown in Figure 4c,d, while the proposed method gives 11 keypoint matches in Figure 4e, and nine matches are visually correct. Visually, the SIFT main orientation and the proposed method give almost the same correct rate of matches, except that the proposed method gives more matches. The reason might be that although this pair of images was taken with a visible camera and an infrared camera, they are very close to single-spectrum images, *i.e.*, brighter (darker) areas in the visible image are also brighter (darker) in the infrared image. However, the relationship between image intensities is not linear, which makes COM and HOI not perform very well.

**Figure 5:**
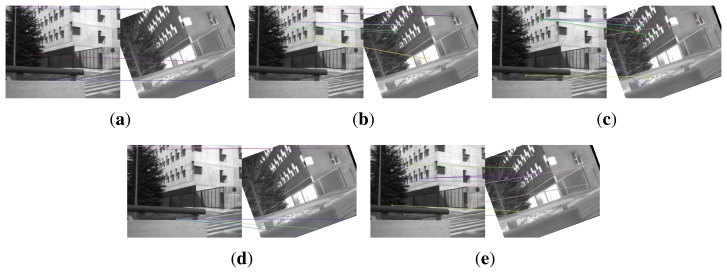
The matched keypoints built with descriptors. (**a**) The original EOH without main orientation; (**b**) the main orientation computed by SIFT, ranging from [0, 2*π*]; (**c**) the main orientation computed by COM; (**d**) the main orientation computed by HOI; (**e**) the proposed method that utilizes the main orientation computed by PIIFD. The test (IR) image is rotated by 20°.

Figure 5 illustrates the keypoint matches on an image pair from dataset VS-LWIR built with EOH, EOH equipped with SIFT main orientation, COM main orientation, HOI main orientation and the proposed method. The performance of EOH and EOH equipped with SIFT main orientation in Figure 5a,b is inferior to that in Figure 4a,b. The performance of COM and HOI is not good either, as shown in Figure 5c,d. This image pair is taken with a visible camera and an LWIR camera. The multimodality between them causes the inaccuracy of SIFT main orientation, COM and HOI and, hence, the mismatches in Figure 5b–d. The proposed method, for the induction of main orientation to keypoints, performs still well on this image pair.

### 4.2. Quantitative Results

This section presents quantitative results. The above visual results can only provide a simpler comparison on a few image pairs, and the comparison result is affected by an individual criterion on “correct” matches. To quantitatively assess the performance for different methods, we perform statistics on the number of correct matches. We define a keypoint match to be correct if the distance *d* between the two keypoints comprising the match is smaller than a threshold *d*_0_. In the literature, different values have been used for *d*_0_, e.g., *d*_0_ = 2, *d*_0_ = 4, *d*_0_ = 5, *etc*. [4]. To eliminate the effect of *d*_0_ on the performance comparison, *d*_0_ = 1, 2, 3, 4, 5, 10, 20, 50, 100 are used in this work. The number of keypoint matches of distance *d* < *d*_0_ is counted and listed in Table 1 for dataset EOIR and Table 2 for dataset VS-LWIR.

In Tables 1 and 2, the test (infrared) image is rotated by 10°, 20°, 30° and 45°. The proposed method outperforms the original EOH for all rotation degrees. For example, when the test image is rotated by 10°, 45.60% of the keypoint matches on dataset EOIR built with the proposed method has a distance less than five, *i.e.*, falling in the range [0, 5], while the EOH has 33.17% falling in [0, 5]. On dataset VS-LWIR, the proposed method has 29.91% matches falling in [0, 5], while the EOH has 19.53%. Additionally, this also indicates that the dataset VS-LWIR is more challenging than EOIR. Both the proposed method and EOH provide superior results on dataset EOIR over VS-LWIR. COM and HOI perform only slightly better than the original EOH without the main orientation on EOIR and VS-LWIR. This to some extent indicates that the main orientations computed with COM and HOI on visible and infrared images are not so accurate as the ones computed with PIIFD, failing to account for the rotation difference between two images.

**Figure 6:**
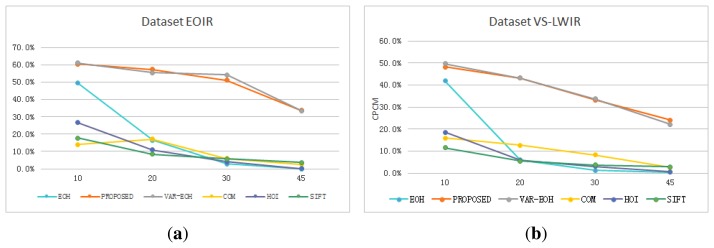
Comparison of keypoint matches for different methods. The horizontal axis represents the degree of rotation, and the vertical axis represents the percent of correct matches. Correct matches are defined to be of a distance falling in (0 10]. (**a**) The result on dataset EOIR; (**b**) the result on dataset VS-long-wave infrared (LWIR). On both EOIR and VS-LWIR, the performance of every method decreases when the rotation degree increases. The proposed method and the variant EOH with main orientation decrease significantly slower than the original EOH, SIFT main orientation, COM and HOI on both datasets, varying the effectiveness of the main orientation.

The performance decreases with the increase of rotation degree for all methods. For example, on dataset VS-LWIR, when the test image is rotated by 10°, the proposed method has 48.13% of matches falling in [0, 10], but this number decreases to 43.08%, 33.13% and 24.02% when the test image is rotated by 20°, 30° and 45°. For EOH, the percent of keypoint matches falling in [0, 10] decreases more than the proposed method, from 41.83% to 5.93%, 1.20% and 0.44%. The performance decrease for EOH is due to the lack of main orientation, while the decrease for the proposed method originates from the inaccuracy of computing main orientation. Figure 6 shows the performance of different methods under rotation. Keypoint matches of distance *d* ≤ 10 are defined to be correct. From Figure 6, it can be seen that the percent of correct matches for all methods decreases with the increase of rotation degree. On both EOIR and VS-LWIR, the proposed method and the variant implementation of EOH decrease slower than the original EOH without the main orientation, SIFT main orientation, COM main orientation and HOI main orientation.

The variant implementation of EOH with the main orientation performs comparable to the proposed method that utilizes the five filters in Figure 1. On dataset VS-LWIR, the variant gives 18.55% of matches falling in [0, 5] when the rotation is 20°, and the proposed method gives 16.50%. When the rotation gets to 45°, the variant gives 11.79%, and the proposed method gives 12.35%. It can also be observed from Figure 6 that the variant EOH proposed in Section 2.3 performs as well as the proposed method. From Tables 1 and 2, we can conclude that the proposed variant implementation of EOH can yield keypoint matches as reliable as the EOH assigned to the main orientation. This explains and verifies that the non-direction bin does not have a great effect on the matching ability and is not used in descriptors, such as SIFT, SURF and PIIFD.

## 5. Conclusions

This paper proposed an approach to assigning the main orientation to keypoints. The PIIFD main orientation was calculated for a keypoint, and then, the EOH descriptor is computed with respect to the main orientation. Experimental results show that the assigned main orientation can significantly improve the matching performance of EOH on images of misalignment containing rotation. Additionally, a variant of EOH is proposed that employs the gradient orientation as the filter responses. The variant EOH can be computed with respect to the main orientation more easily and achieve a comparable matching performance to the original EOH, but needs less computational cost.

## Figures and Tables

**Table 1:** Comparison of keypoint matches for different methods on dataset EOIR. The cumulative percent of correct matches (CPCM) is presented for different *d*_0_.

**Rotation**	**Error**	**[0 1]**	**(1 2]**	**(2 3]**	**(3 4]**	**(4 5]**	**(5 10]**	**(10 20]**	**(20 50]**	**(50 100]**	**>100**
10°	Proposed	142	134	98	50	48	153	99	56	59	196
	CPCM (%)	13.72	26.67	36.14	40.97	45.60	60.39	69.95	75.36	81.06	100.00

	EOH	74	73	55	37	32	132	134	59	75	146
	CPCM (%)	9.06	17.99	24.72	29.25	33.17	49.33	65.73	72.95	82.13	100.00

	COM	17	15	11	10	6	63	65	103	142	437
	CPCM (%)	1.96	3.68	4.95	6.10	6.79	14.04	21.52	33.37	49.71	100.00

	HOI	32	22	14	14	16	104	110	89	123	236
	CPCM (%)	4.21	7.11	8.95	10.79	12.89	26.58	41.05	52.76	68.95	100.00

	VAR-EOH	143	131	99	60	51	155	69	69	55	216
	CPCM (%)	13.65	26.15	35.59	41.32	46.18	60.97	67.56	74.14	79.39	100.00

20°	Proposed	97	131	83	70	54	112	83	49	72	203
	CPCM (%)	10.17	23.90	32.60	39.94	45.60	57.34	66.04	71.17	78.72	100.00

	EOH	10	8	11	2	4	40	52	84	69	176
	CPCM (%)	2.19	3.95	6.36	6.80	7.68	16.45	27.85	46.27	61.40	100.00

	COM	20	26	22	14	22	77	105	148	182	448
	CPCM (%)	1.88	4.32	6.39	7.71	9.77	17.01	26.88	40.79	57.89	100.00

	HOI	6	12	8	12	16	84	120	179	307	535
	CPCM (%)	0.47	1.41	2.03	2.97	4.22	10.79	20.17	34.17	58.17	100.00

	VAR-EOH	101	129	75	63	49	103	71	74	75	199
	CPCM (%)	10.76	24.49	32.48	39.19	44.41	55.38	62.94	70.82	78.81	100.00

30°	Proposed	85	121	71	51	32	115	81	55	84	234
	CPCM (%)	9.15	22.17	29.82	35.31	38.75	51.13	59.85	65.77	74.81	100.00

	EOH	0	0	1	0	0	8	16	48	50	194
	CPCM (%)	0.00	0.00	0.32	0.32	0.32	2.84	7.89	23.03	38.80	100.00

	COM	1	4	5	4	4	43	64	135	214	564
	CPCM (%)	0.10	0.48	0.96	1.35	1.73	5.88	12.04	25.05	45.66	100.00

	HOI	0	1	1	1	0	11	9	47	52	224
	CPCM (%)	0.00	0.29	0.58	0.87	0.87	4.05	6.65	20.23	35.26	100.00

	VAR-EOH	90	115	76	68	45	113	89	51	77	213
	CPCM (%)	9.61	21.88	29.99	37.25	42.05	54.11	63.61	69.05	77.27	100.00

45°	Proposed	61	87	43	38	31	100	54	58	137	463
	CPCM (%)	5.69	13.81	17.82	21.36	24.25	33.58	38.62	44.03	56.81	100.00

	EOH	0	0	0	0	0	0	2	22	53	406
	CPCM (%)	0.00	0.00	0.00	0.00	0.00	0.00	0.41	4.97	15.94	100.00

	COM	0	2	1	3	3	33	52	105	183	1200
	CPCM (%)	0.00	0.13	0.19	0.38	0.57	2.65	5.94	12.58	24.15	100.00

	HOI	0	0	0	0	0	1	3	21	95	469
	CPCM (%)	0.00	0.00	0.00	0.00	0.00	0.17	0.68	4.24	20.37	100.00

	VAR-EOH	53	74	56	46	34	116	68	57	167	463
	CPCM (%)	4.67	11.20	16.14	20.19	23.19	33.42	39.42	44.44	59.17	100.00

**Table 2:** Comparison of keypoint matches for different methods on dataset VS-LWIR. The cumulative percent of correct matches (CPCM) is presented.

**Rotation**	**Error**	**[0 1]**	**(1 2]**	**(2 3]**	**(3 4]**	**(4 5]**	**(5 10]**	**(10 20]**	**(20 50]**	**(50 100]**	**>100**
10°	Proposed	16	65	81	50	44	156	109	82	50	203
	CPCM (%)	1.87	9.46	18.93	24.77	29.91	48.13	60.86	70.44	76.29	100.00

	EOH	9	27	41	37	27	161	146	70	39	165
	CPCM (%)	1.25	4.99	10.66	15.79	19.53	41.83	62.05	71.75	77.15	100.00

	COM	8	19	13	15	11	86	118	123	112	450
	CPCM (%)	0.84	2.83	4.19	5.76	6.91	15.92	28.27	41.15	52.88	100.00

	HOI	8	11	5	9	17	79	124	94	73	280
	CPCM (%)	1.14	2.71	3.43	4.71	7.14	18.43	36.14	49.57	60.00	100.00

	VAR-EOH	17	66	73	70	40	162	106	71	41	216
	CPCM (%)	1.97	9.63	18.10	26.22	30.86	49.65	61.95	70.19	74.94	100.00

20°	PROPOSED	22	44	52	32	46	162	136	65	42	230
	CPCM (%)	2.65	7.94	14.20	18.05	23.59	43.08	59.45	67.27	72.32	100.00

	EOH	0	0	4	2	3	21	33	46	39	358
	CPCM (%)	0.00	0.00	0.79	1.19	1.78	5.93	12.45	21.54	29.25	100.00

	COM	3	5	4	5	14	93	94	128	176	458
	CPCM (%)	0.31	0.82	1.22	1.73	3.16	12.65	22.24	35.31	53.27	100.00

	HOI	0	4	8	12	7	50	103	163	211	836
	CPCM (%)	0.00	0.29	0.86	1.72	2.22	5.81	13.20	24.89	40.03	100.00

	VAR-EOH	21	47	40	37	47	159	126	69	41	227
	CPCM (%)	2.58	8.35	13.27	17.81	23.59	43.12	58.60	67.08	72.11	100.00

30°	Proposed	7	31	31	33	34	137	105	88	37	321
	CPCM (%)	0.85	4.61	8.37	12.38	16.50	33.13	45.87	56.55	61.04	100.00

	EOH	0	0	0	0	0	9	4	32	62	642
	CPCM (%)	0.00	0.00	0.00	0.00	0.00	1.20	1.74	6.01	14.29	100.00

	COM	0	2	9	13	6	69	102	106	127	771
	CPCM (%)	0.00	0.17	0.91	1.99	2.49	8.22	16.68	25.48	36.02	100.00

	HOI	1	0	0	0	1	12	19	48	53	337
	CPCM (%)	0.21	0.21	0.21	0.21	0.42	2.97	7.01	17.20	28.45	100.00

	VAR-EOH	8	25	51	35	29	121	112	85	55	277
	CPCM (%)	1.00	4.14	10.53	14.91	18.55	33.71	47.74	58.40	65.29	100.00

45°	Proposed	10	26	24	23	27	104	8	86	52	450
	CPCM (%)	1.12	4.04	6.73	9.32	12.35	24.02	34.01	43.66	49.49	100.00

	EOH	1	0	0	0	0	4	8	42	127	967
	CPCM (%)	0.09	0.09	0.09	0.09	0.09	0.44	1.13	4.79	15.84	100.00

	COM	1	0	3	7	3	33	59	109	161	1448
	CPCM (%)	0.05	0.05	0.22	0.60	0.77	2.58	5.81	11.79	20.61	100.00

	HOI	1	0	0	0	0	2	12	49	76	347
	CPCM (%)	0.21	0.21	0.21	0.21	0.21	0.62	3.08	13.14	28.75	100.00

	VAR-EOH	9	24	23	25	27	94	98	70	66	480
	CPCM (%)	0.98	3.60	6.11	8.84	11.79	22.05	32.75	40.39	47.60	100.00
